# Monocyte-to-High-Density-Lipoprotein Ratio Is Associated with Ventricular Arrhythmia in Patients with Implantable Cardioverter-Defibrillators: A Cross-Sectional Study

**DOI:** 10.3390/jcdd13090420

**Published:** 2026-08-28

**Authors:** Murat Küçükukur, Cenk Ekmekçi

**Affiliations:** Department of Cardiology, İzmir City Hospital, İzmir 35530, Türkiye; cenk.ekmekci@saglik.gov.tr

**Keywords:** monocyte-to-HDL ratio, ventricular arrhythmia, implantable cardioverter-defibrillator, inflammation, sudden cardiac death, risk stratification

## Abstract

Background: Residual arrhythmic risk persists in recipients of implantable cardioverter-defibrillators (ICDs) despite guideline-based selection, and inexpensive markers that refine this risk are needed. The monocyte-to-high-density-lipoprotein ratio (MHR) integrates a pro-inflammatory (monocyte) and an anti-inflammatory/antioxidant (HDL) signal in a single index derived from routine blood work. We examined whether MHR is associated with ventricular arrhythmia in ICD patients. Methods: In this cross-sectional study we assessed 382 ICD recipients attending a device outpatient clinic and classified them by a composite ventricular arrhythmia outcome (non-sustained ventricular tachycardia, sustained ventricular tachycardia, or ventricular fibrillation) documented on device interrogation. MHR and comparator inflammatory markers were measured at the same visit. Discrimination was assessed by receiver operating characteristic (ROC) analysis, the optimal threshold by the Youden index, and independence by multivariable logistic regression; internal validity was examined with repeated cross-validation, bootstrap optimism correction, and calibration testing. Results: The ventricular arrhythmia outcome was present in 58 patients (15.2%). MHR was higher in patients with the outcome (median 16.3 vs. 12.3, *p* < 0.001) and discriminated it with an area under the curve (AUC) of 0.756 (95% CI 0.673–0.828). At the optimal threshold of 15.71, sensitivity was 63.8% and specificity was 83.3%, with a negative predictive value of 92.8%; the outcome was present in 40.7% of patients above versus 7.2% below this cut-off. MHR remained independently associated with the outcome after adjustment for age, left ventricular ejection fraction, etiology, and renal function (odds ratio 1.23 per unit, 95% CI 1.16–1.32; *p* < 0.001) and outperformed high-sensitivity C-reactive protein (DeLong *p* < 0.001). Discrimination was preserved in both ischaemic and non-ischaemic cardiomyopathy without interaction by etiology (*p* = 0.67). The model was stable on internal validation (cross-validated AUC 0.761; optimism 0.021; Hosmer–Lemeshow *p* = 0.66). Conclusions: MHR is independently associated with documented ventricular arrhythmia in ICD patients and, being derived from routine tests, may warrant prospective evaluation as a low-cost adjunct for risk stratification.

## 1. Introduction

Sudden cardiac death remains a leading cause of cardiovascular mortality, and the implantable cardioverter-defibrillator (ICD) is the cornerstone of both primary and secondary prevention in patients at risk of life-threatening ventricular arrhythmia [1,2]. Landmark trials in ischaemic and non-ischaemic cardiomyopathy established the device’s survival benefit [3,4], although the more recent DANISH trial tempered expectations in the non-ischaemic setting and indicated that not all candidates derive equal benefit [5]. Current selection still rests largely on left ventricular ejection fraction, a criterion that is neither sensitive nor specific for the individual patient, so that many recipients never receive appropriate therapy while some deaths occur in patients who would not have qualified [1,2,5].

This imprecision matters clinically because device therapy is not benign: appropriate and inappropriate shocks are themselves associated with worsening heart failure and increased mortality [6]. Refining arrhythmic risk beyond ejection fraction, ideally with markers that are inexpensive, reproducible, and already embedded in routine care, has therefore become an active priority, and inflammatory indices have attracted particular interest as candidate predictors of ventricular arrhythmia and sudden death [7]. The rationale is mechanistic rather than incidental. Systemic inflammation promotes electrical instability through ion-channel remodeling, gap junction dysfunction, and autonomic imbalance, and chronic inflammatory signaling contributes to the fibrotic substrate on which re-entrant arrhythmias depend [8]. Monocytes and their macrophage progeny are central to this process: they infiltrate the injured and remodeling myocardium, orchestrate scar formation, and modulate fibroblast activity, linking the circulating monocyte pool to the structural arrhythmic substrate [9,10,11].

High-density lipoprotein (HDL) exerts a counter-regulatory influence, with well-characterized anti-inflammatory, antioxidant, and endothelial-protective properties that oppose monocyte activation and oxidative injury [12,13,14]. The monocyte-to-HDL ratio (MHR) was conceived to capture the balance between these opposing forces in a single number obtainable from a routine complete blood count and lipid panel [15]. Since its original description in chronic kidney disease [15], MHR has been validated as a prognostic marker across the cardiovascular spectrum [16,17]: it tracks coronary disease severity and predicts major adverse events in acute coronary syndromes [18], relates to the no-reflow phenomenon after primary intervention [19], and forecasts mortality after myocardial infarction and percutaneous coronary intervention [20,21,22]. In the electrophysiological domain, a higher pre-procedural MHR predicts atrial fibrillation recurrence after ablation [23,24], relates to device-detected atrial high-rate episodes [25], and an elevated MHR has been linked to adverse outcomes in heart failure [26], reinforcing the notion that the ratio indexes an arrhythmogenic inflammatory milieu.

Evidence connecting MHR to ventricular arrhythmia, however, is sparse. The closest available data show an association between MHR and frequent premature ventricular complexes [27], and inflammatory surrogates such as the neutrophil-to-lymphocyte ratio and C-reactive protein have been related to appropriate ICD therapy and ventricular events in selected populations [28,29,30]. To our knowledge, no study has evaluated MHR specifically in relation to ventricular arrhythmia in an ICD cohort. We therefore tested the hypothesis that MHR is independently associated with device-documented ventricular arrhythmia in ICD recipients, sought an optimal discriminatory threshold, compared its performance against established inflammatory markers, and examined whether this association differs between ischaemic and non-ischaemic cardiomyopathy.

## 2. Materials and Methods

### 2.1. Study Population

This cross-sectional observational study included consecutive patients attending the cardiac device outpatient clinic who had previously undergone implantation of an ICD or cardiac resynchronization defibrillator for primary or secondary prevention according to prevailing guideline indications [1,2]. Patients were eligible if they were at least 18 years of age and had a functioning device with complete laboratory and clinical data at the index visit. Patients with active infection, known malignancy, chronic inflammatory or hematological disease, or recent (within one month) acute coronary syndrome were excluded so that baseline inflammatory indices reflected the chronic cardiac substrate rather than an intercurrent process. The study conformed to the Declaration of Helsinki, was approved by the institutional ethics committee, and all participants provided written informed consent.

### 2.2. Data Collection and Laboratory Measurements

Demographic, clinical, echocardiographic, and pharmacological data were recorded at the index visit, including age, sex, left ventricular ejection fraction, cardiomyopathy etiology, device indication and type, comorbidities, and guideline-directed medical therapy. Cardiomyopathy etiology was classified as ischaemic if the patient had angiographically documented obstructive coronary artery disease, a prior myocardial infarction, or previous coronary revascularisation, and as non-ischaemic otherwise. Venous blood was drawn at the same visit before device interrogation. Complete blood count, lipid profile, renal and hepatic panels, high-sensitivity C-reactive protein (hs-CRP), and N-terminal pro-B-type natriuretic peptide were measured by standard automated methods. Specifically, complete blood counts were performed on a Sysmex XN-series automated hematology analyzer (Sysmex Corporation, Kobe, Japan), and serum biochemical parameters, including the lipid profile, hs-CRP, and N-terminal pro-B-type natriuretic peptide, were measured on a Roche Cobas modular analyzer platform (Roche Diagnostics, Mannheim, Germany). The estimated glomerular filtration rate (eGFR) was calculated with the Chronic Kidney Disease Epidemiology Collaboration (CKD-EPI) creatinine equation. MHR was calculated as the absolute peripheral blood monocyte count divided by the serum HDL-cholesterol concentration; the neutrophil-to-lymphocyte ratio (NLR) was calculated as the absolute neutrophil count divided by the absolute lymphocyte count. Investigators performing laboratory measurements were blinded to clinical outcomes.

### 2.3. Endpoint Definition

The outcome of interest was the presence of ventricular arrhythmia on device interrogation at the index visit, defined as a composite of non-sustained ventricular tachycardia (NSVT) and device-treated ventricular tachycardia or ventricular fibrillation (VT/VF) recorded since the patient’s previous scheduled clinic visit; routine follow-up at our device clinic is scheduled at approximately six-month intervals, so recorded episodes generally reflect events accumulated over this period. VT and VF episodes were defined as those that received appropriate device therapy (antitachycardia pacing or shock); NSVT was defined as a run of ventricular beats that terminated spontaneously without device therapy. Arrhythmic episodes were adjudicated from stored device electrograms by two electrophysiologists blinded to laboratory values; disagreements were resolved by consensus. Detailed device programming parameters (for example, detection zone cut-offs and antitachycardia pacing algorithms) were not systematically recorded as part of the study protocol and could not be analyzed.

### 2.4. Statistical Analysis

The sample size was estimated a priori in the study protocol using Schoenfeld’s events-based method, assuming at least a two-fold increase in arrhythmic risk in the high-MHR group, a two-sided α of 0.05, statistical power of 80%, an anticipated event proportion of 30%, and a 10% allowance for incomplete data, which yielded a minimum requirement of approximately 240 patients. The final cohort of 382 consecutive patients exceeded this requirement, and the 58 observed events provided more than ten events per candidate covariate for the five-covariate multivariable model, in accordance with accepted recommendations. Continuous variables are summarized as median (interquartile range) and compared with the Mann–Whitney U test, since most were non-normally distributed; categorical variables are expressed as counts (percentages) and compared with the chi-squared test, or Fisher’s exact test where an expected cell count was below 5. The discriminative performance of MHR for the primary outcome was evaluated by receiver operating characteristic (ROC) analysis, with the area under the curve (AUC) and its 95% confidence interval estimated by bootstrapping (3000 resamples). The optimal cut-off was identified by the Youden J index, and sensitivity, specificity, predictive values, and accuracy were derived at that threshold; the stability of the threshold was examined by bootstrap resampling. AUCs of MHR, NLR, and hs-CRP were compared with the DeLong test for correlated ROC curves.

The independent association of MHR with the outcome was assessed by multivariable logistic regression including clinically selected covariates (age, left ventricular ejection fraction, ischaemic etiology, and estimated glomerular filtration rate) chosen a priori and constrained to respect the number of events. Because arrhythmia status was ascertained at a single time point without individual event dates, a time-to-event (Cox) analysis was not applicable. Results are reported as odds ratios (OR) with 95% confidence intervals. Multicollinearity was assessed by variance inflation factors, and the robustness of the MHR estimate was tested in sensitivity models further adjusted for diabetes, hypertension, device indication (primary versus secondary prevention), device type (ICD versus CRT-D), statin therapy, amiodarone use, and mineralocorticoid-receptor-antagonist (MRA) use, factors that could plausibly influence monocyte activity, HDL metabolism, or arrhythmic risk directly. ACE inhibitor, angiotensin receptor blocker, or angiotensin receptor–neprilysin inhibitor (ACEI/ARB/ARNI) therapy was examined descriptively rather than entered as a covariate, because its distribution relative to the outcome (below) precluded stable estimation. Model performance was internally validated by 20 repetitions of five-fold cross-validation and by bootstrap optimism correction, and calibration was assessed with the Hosmer–Lemeshow test. Subgroup discrimination was examined separately in ischaemic and non-ischaemic cardiomyopathy, with a multiplicative interaction term testing effect modification by etiology. A two-sided *p* < 0.05 was considered significant. Baseline comparisons (Mann–Whitney U, chi-squared, and Fisher’s exact tests), multivariable logistic regression with variance inflation factor collinearity diagnostics, and the Hosmer–Lemeshow calibration test were performed in IBM SPSS Statistics, version 25 (IBM Corp., Armonk, NY, USA). Receiver operating characteristic analysis with bootstrap confidence intervals and threshold stability resampling, the DeLong test for comparison of correlated ROC curves, and internal validation by repeated five-fold cross-validation and bootstrap optimism correction were performed in Python version 3.12 (statsmodels and scikit-learn).

Of 383 patients initially assessed, one was excluded for incomplete outcome ascertainment, leaving 382 for analysis. All variables entering the primary multivariable model (MHR, age, left ventricular ejection fraction, etiology, estimated glomerular filtration rate) were complete for these 382 patients. Missingness in secondary laboratory variables not included in the primary model was low (glycated hemoglobin 7.9%, calcium 2.4%, magnesium 3.4%, N-terminal pro-B-type natriuretic peptide < 1%) and was handled by pairwise complete case exclusion in the relevant descriptive comparisons; no imputation was performed.

## 3. Results

### 3.1. Baseline Characteristics

The study comprised 382 ICD recipients, of whom 58 (15.2%) had a device-documented ventricular arrhythmia outcome. Baseline characteristics stratified by outcome are shown in Table 1. Patients who developed arrhythmia were older (median 70 vs. 66 years, *p* = 0.028) and more frequently had ischaemic cardiomyopathy, diabetes, and hypertension, and they more often carried a secondary prevention indication. The two groups had comparable left ventricular ejection fraction and N-terminal pro-B-type natriuretic peptide concentrations. They were also more intensively treated with heart failure-directed medical therapy: mineralocorticoid receptor antagonist use was more common among patients with the outcome (74.1% vs. 49.7%, *p* = 0.001), and every patient with the outcome was receiving an ACE inhibitor, angiotensin receptor blocker, or angiotensin receptor–neprilysin inhibitor, compared with 85.2% of those without (*p* = 0.004).

**Figure 1:**
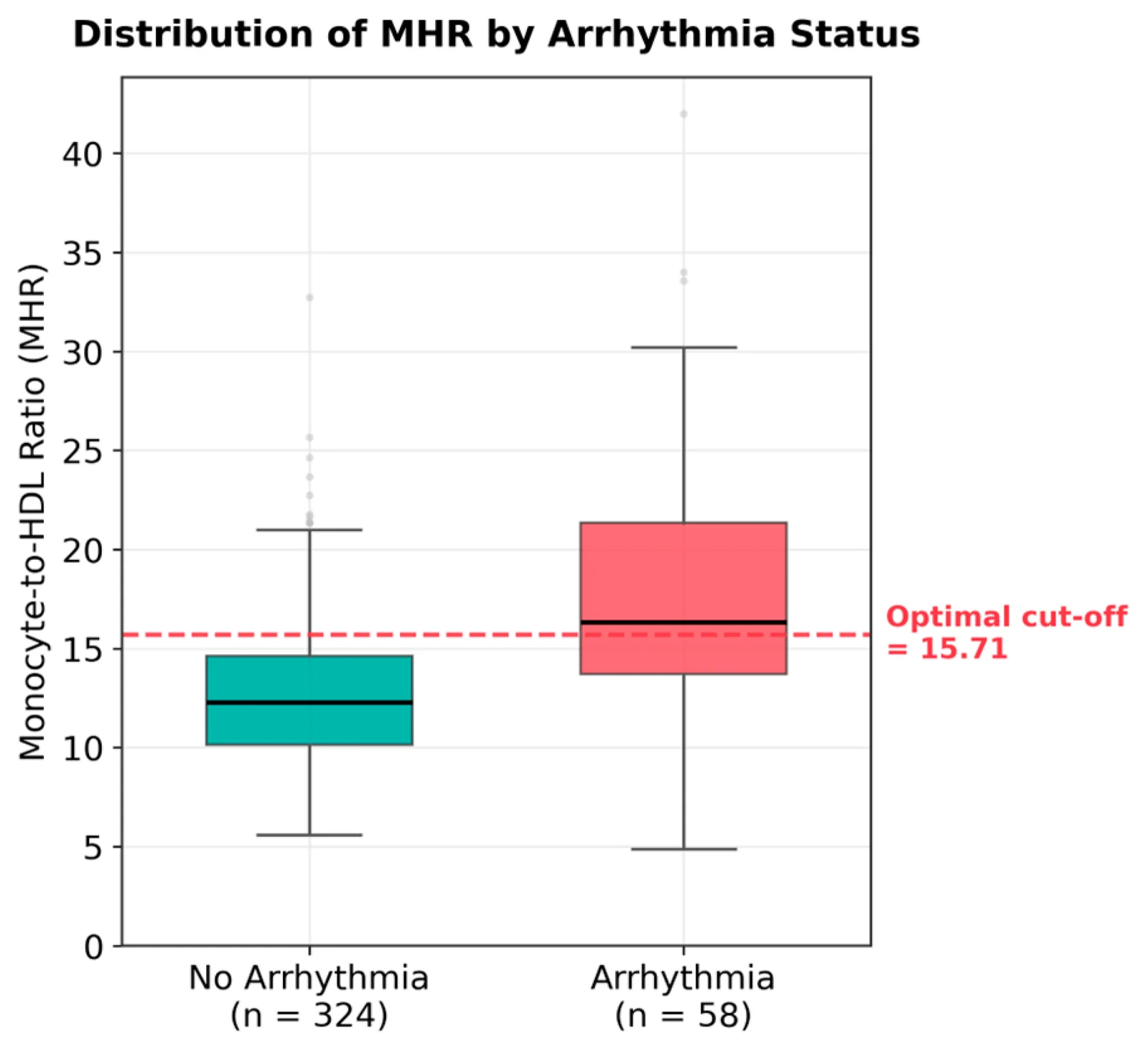
Distribution of the monocyte-to-HDL ratio (MHR) by arrhythmia status. Boxes show the median and interquartile range; the dashed line marks the optimal cut-off of 15.71.

The hematological profile clearly distinguished the groups. Total leukocyte, neutrophil, and monocyte counts were all higher among patients with events, whereas HDL-cholesterol was essentially identical (median 44 mg/dL in both groups); the difference in MHR was therefore driven by the monocyte component rather than by HDL. Median MHR was 16.3 (13.7–21.3) in patients with arrhythmia versus 12.3 (10.1–14.6) in those without (*p* < 0.001; Figure 1), and NLR was likewise elevated (2.84 vs. 2.15, *p* < 0.001). By contrast, hs-CRP did not differ between groups (*p* = 0.85).

### 3.2. Discriminative Performance and Optimal Threshold

**Figure 2:**
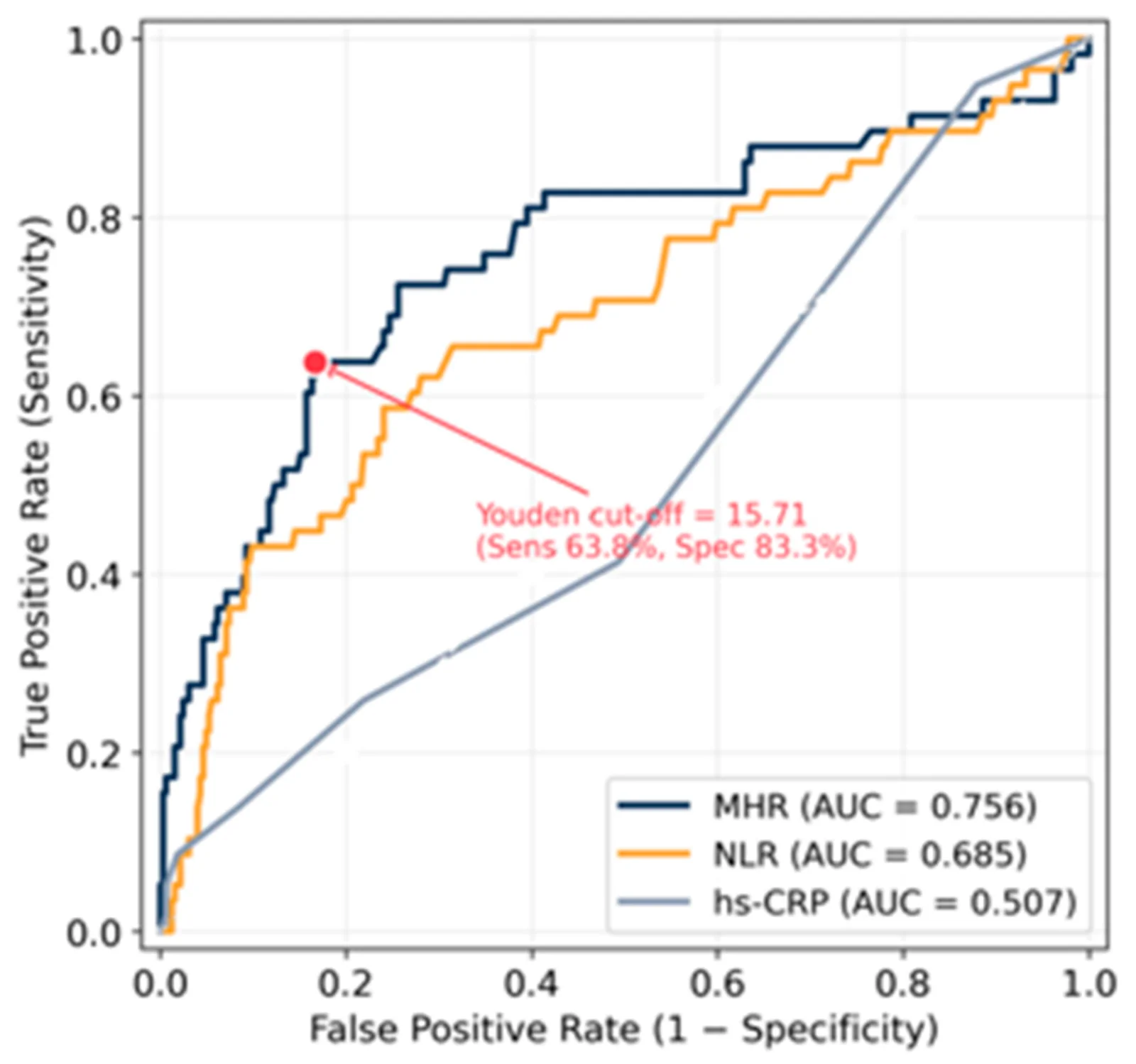
Receiver operating characteristic curves for MHR, the neutrophil-to-lymphocyte ratio (NLR), and high-sensitivity C-reactive protein (hs-CRP) for the ventricular arrhythmia outcome. The marked point indicates the Youden-optimal MHR threshold.

**Figure 3:**
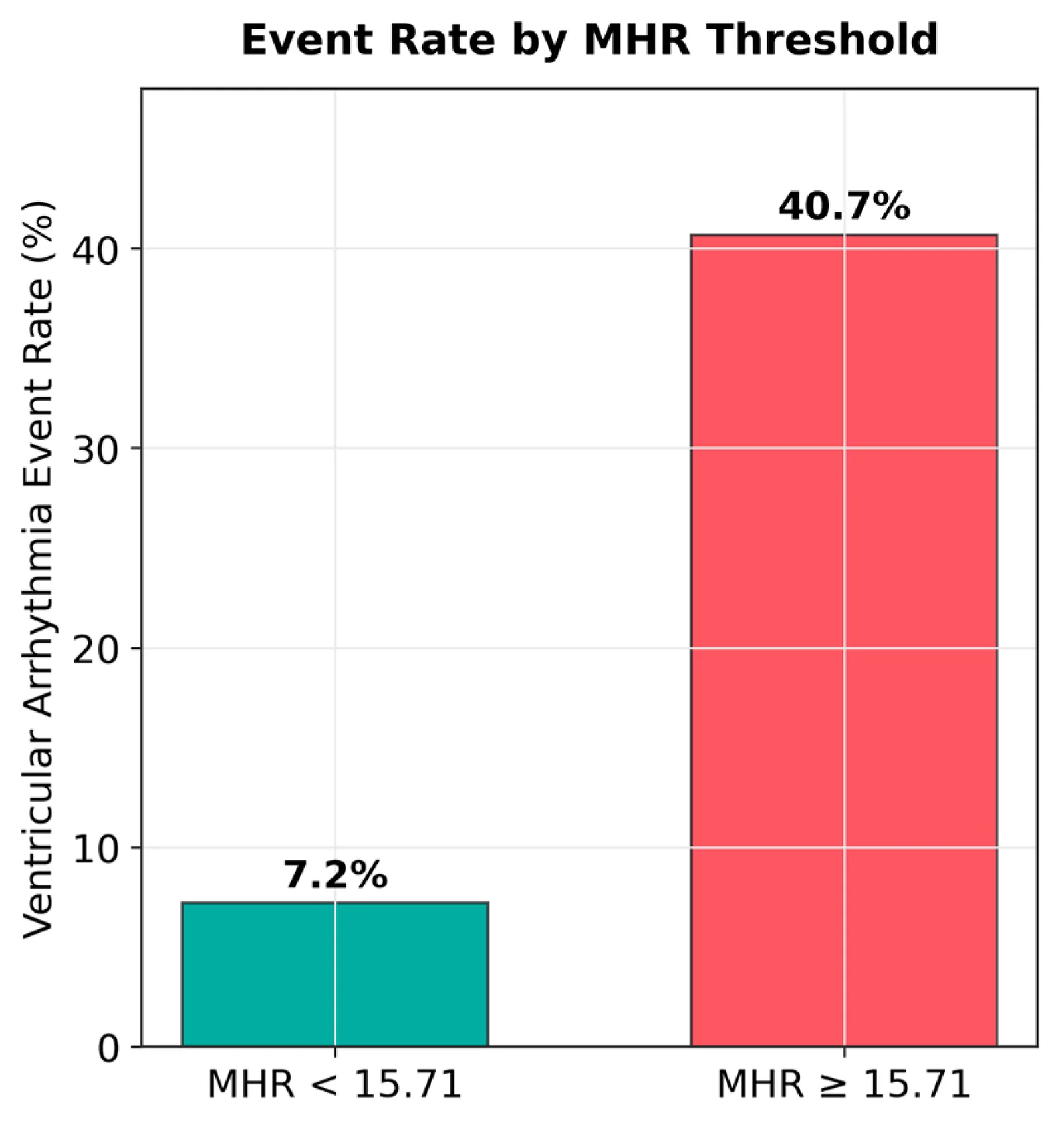
Ventricular arrhythmia event rate below and at or above the optimal MHR threshold of 15.71.

MHR discriminated the ventricular arrhythmia outcome with an AUC of 0.756 (95% CI 0.673–0.828; Figure 2). The Youden-optimal cut-off was 15.71, yielding a sensitivity of 63.8% and specificity of 83.3%, with a positive predictive value of 40.7%, a negative predictive value of 92.8%, and an overall accuracy of 80.4%. The high negative predictive value indicates that arrhythmia was uncommon below the threshold: the event rate was 7.2% in patients with MHR below 15.71 compared with 40.7% in those at or above it (Figure 3). Dichotomized at this threshold, an MHR at or above 15.71 was associated with an approximately nine-fold increase in the odds of the outcome (OR 8.81, 95% CI 4.79–16.21; *p* < 0.001). Bootstrap resampling placed the median optimal threshold at 14.75 (interquartile range 14.5–15.7), consistent with the point estimate.

### 3.3. Comparison with Other Inflammatory Markers

Among the markers examined, MHR provided the strongest discrimination (AUC 0.756), exceeding NLR (0.685) and hs-CRP (0.507). The superiority of MHR over hs-CRP was statistically significant (DeLong *p* < 0.001), whereas the difference versus NLR did not reach significance (*p* = 0.204) despite a numerically higher AUC for MHR. That hs-CRP alone was non-discriminatory in this cohort suggests that the information carried by MHR reflects a dimension of monocyte-driven inflammatory activation distinct from the classical acute-phase response indexed by C-reactive protein [7,30].

### 3.4. Independent Association with Ventricular Arrhythmia

**Figure 4:**
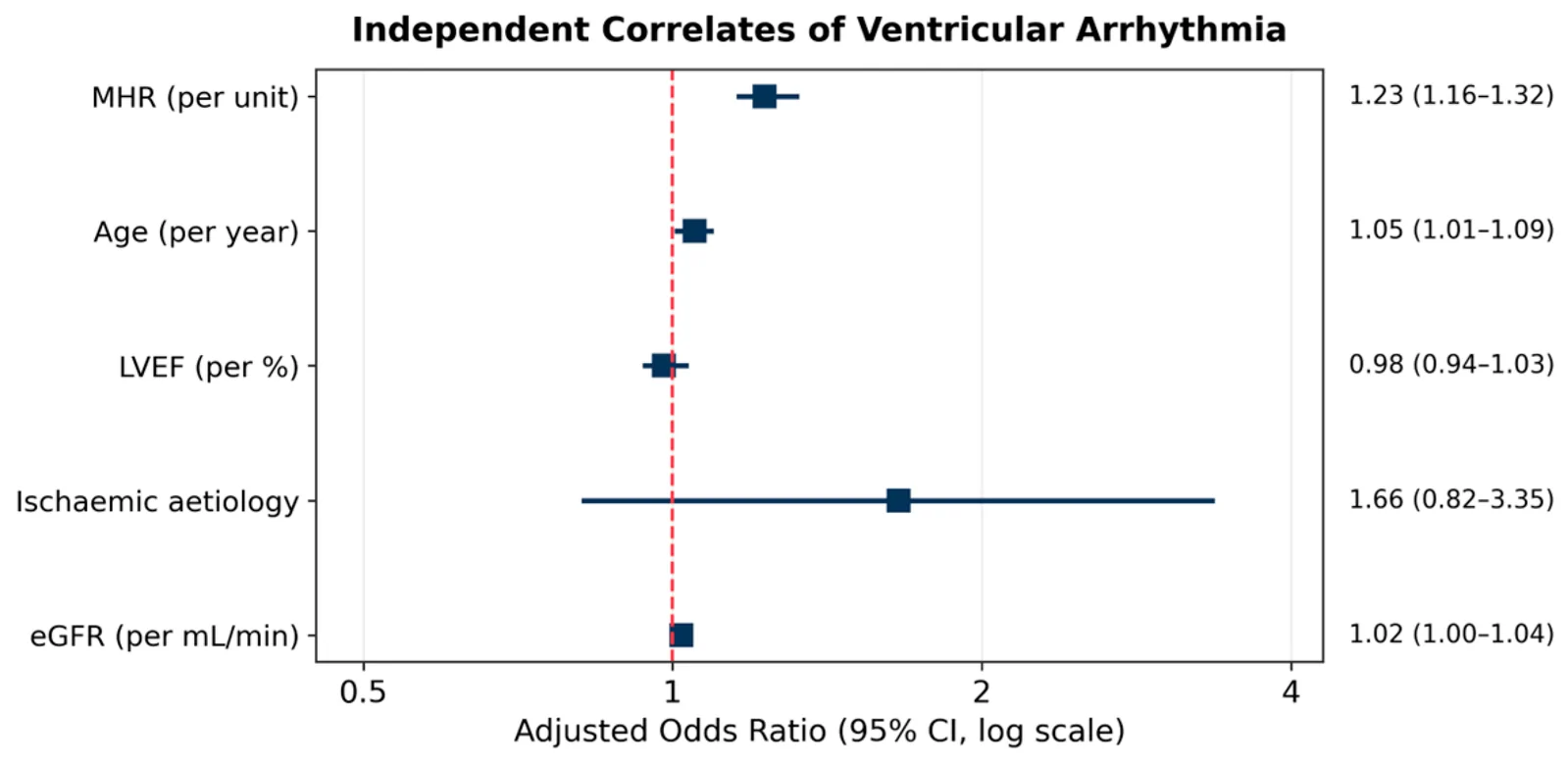
Adjusted odds ratios (95% confidence intervals) for the independent correlates of the ventricular arrhythmia outcome in the multivariable logistic-regression model.

In univariable logistic regression, each one-unit increase in MHR was associated with a 22% increase in the odds of the outcome (OR 1.22, 95% CI 1.15–1.30; *p* < 0.001). This association was essentially unchanged after multivariable adjustment for age, left ventricular ejection fraction, ischaemic etiology, and estimated glomerular filtration rate (adjusted OR 1.23 per unit, 95% CI 1.16–1.32; *p* < 0.001; Table 2), with the model having an AUC of 0.780 (Figure 4). Older age and higher estimated glomerular filtration rate were also independently associated with the outcome, whereas ejection fraction was not; estimated glomerular filtration rate was not associated with the outcome univariably (OR 1.011, 95% CI 0.996–1.027; *p* = 0.156) and reached significance only after adjustment, chiefly for age, with which it was inversely correlated (Spearman r = −0.25). The between-group difference in eGFR in Table 1 was similarly non-significant (*p* = 0.067), so the apparent independent association emerged only on multivariable adjustment. Variance inflation factors were near unity for all covariates, indicating no meaningful collinearity, and the MHR estimate was stable when the model was further adjusted for diabetes, hypertension, device indication, statin therapy, amiodarone use, device type (ICD versus CRT-D), and MRA use (adjusted OR 1.22–1.26 across sensitivity models; *p* < 0.001 in each). Neither statin therapy nor amiodarone use was itself independently associated with the outcome (*p* = 0.47 and *p* = 0.42, respectively), and MHR was not correlated with statin use (Spearman r = 0.02); MRA use, in contrast, was itself independently associated with the outcome in this sensitivity model (adjusted OR 4.64, *p* < 0.001), consistent with its role as a marker of more advanced heart failure, but did not attenuate the MHR association. ACEI/ARB/ARNI therapy could not be entered as a covariate: every one of the 58 patients with the outcome was receiving one of these agents, compared with 85.2% of patients without it (Table 1), and this complete separation prevented a stable coefficient estimate, so the association is reported descriptively only. Device type showed an apparent inverse association with the outcome in this covariate-adjusted sensitivity model: patients with a CRT-D had lower odds of the outcome than those with a conventional ICD (adjusted OR 0.22, *p* = 0.007), consistent with the lower unadjusted event rate in the CRT-D group (Table 1). This estimate rests on only four arrhythmic events among the 78 CRT-D recipients, however, and is therefore statistically imprecise and should be interpreted with caution rather than as a robust independent effect.

### 3.5. Subgroup Analysis by Etiology

MHR retained its discriminative value in both aetiological subgroups, with an AUC of 0.757 in ischaemic cardiomyopathy (*n* = 255, 43 events) and 0.764 in non-ischaemic cardiomyopathy (*n* = 127, 15 events). The multiplicative interaction between MHR and etiology was not significant (*p* = 0.67), indicating that the discriminative performance of MHR did not depend materially on the underlying substrate.

### 3.6. Internal Validation and Calibration

Internal validation indicated little overfitting. The apparent AUC of 0.780 was closely reproduced by repeated five-fold cross-validation (0.761), and bootstrap optimism was small (0.021), yielding an optimism-corrected AUC of 0.758. MHR alone was equally stable across cross-validation folds (0.756). There was no evidence of miscalibration (non-significant Hosmer–Lemeshow statistic, *p* = 0.66).

## 4. Discussion

In this cross-sectional study of 382 ICD recipients, MHR was independently associated with device-documented ventricular arrhythmia, with moderate discrimination that held after covariate adjustment and remained stable on internal validation. This appears to be the first evaluation of MHR in relation to ventricular arrhythmia in an ICD population, extending a literature that until now reached only as far as premature ventricular complexes [27] and related inflammatory surrogates [28,29,30].

These findings are consistent with the mechanistic rationale outlined above: a higher circulating monocyte burden, set against a diminished anti-inflammatory HDL capacity [12,13,14], plausibly reflects a myocardium primed for electrical instability through the combined effects of monocyte-driven structural remodeling [9,10,11] and the ion-channel and gap junction derangements that accompany chronic inflammation [8]. That the group difference in MHR was driven by the monocyte numerator rather than by HDL (Table 1) mirrors the mechanistic framing under which the ratio was originally proposed [15,16]. Expressing this signal as a ratio to HDL, rather than as the monocyte count alone, is nonetheless conceptually deliberate: MHR is intended to capture the balance between pro-inflammatory monocyte activity and the anti-inflammatory, antioxidant capacity of HDL [12,13,14], a construct that a monocyte count in isolation cannot represent, even in a sample such as ours where HDL itself did not differ by outcome. Our findings thus align with the broader body of work in which MHR predicts adverse outcomes across coronary disease, structural heart disease, and atrial arrhythmia [18,19,20,21,22,23,24,26], now extended to the ventricular domain.

MHR outperformed both NLR and hs-CRP, significantly so for the latter, and hs-CRP did not discriminate the outcome in this cohort. As a stable outpatient population attending a routine device clinic rather than presenting with an acute event, most participants likely had low baseline inflammatory activity, reflected in the narrow distribution of hs-CRP values (median 1.0 mg/L in both groups, Table 1); this restricted range may have limited its discriminative capacity in this setting. Nonetheless, this pattern suggests that MHR captures a monocyte-centered axis of inflammation that only partly overlaps with the neutrophil–lymphocyte balance and is largely independent of the hepatic acute-phase response [7,30]. It also implies that MHR is not simply a generic marker of illness severity, since it added discriminative value where a widely used marker such as hs-CRP did not. Whether MHR and NLR index complementary inflammatory pathways, rather than the same underlying signal at different strengths, warrants further study.

MHR performed comparably in ischaemic and non-ischaemic cardiomyopathy, with no significant interaction by etiology. This matters because the arrhythmic substrates of the two conditions differ, and imaging-based risk markers such as scar burden are typically substrate-specific [31,32]. The etiology-independent performance of MHR suggests that it behaves as a marker of a generalized inflammatory arrhythmogenic diathesis rather than of a scar phenotype confined to ischaemic disease. From a practical standpoint, this broadens its potential applicability, including to the non-ischaemic population in whom conventional risk stratification is especially difficult and in whom the benefit of prophylactic defibrillation remains debated [5,32].

Two further covariate findings warrant brief comment. Estimated glomerular filtration rate did not differ between the groups and was not associated with the outcome univariably (Section 3.4); its weak positive coefficient after adjustment reflects an inverse correlation with age rather than an independent renal mechanism. Patients with a CRT-D, in contrast, had a lower observed arrhythmia burden than those with a conventional ICD despite lower ejection fraction (Table 1), a pattern plausibly related to the reverse electrical and structural remodeling associated with cardiac resynchronization. Because device type was not a prespecified focus of this analysis and the estimate rests on only four arrhythmic events among CRT-D recipients, this observation should be regarded as exploratory and hypothesis-generating.

These observations have potential clinical relevance, with an important caveat about their cross-sectional origin. A practically relevant feature of MHR in our data was its high negative predictive value: only about 7% of patients below the threshold had documented arrhythmia, a group in whom conventional markers such as ejection fraction, which did not differ between patients with and without the outcome (Table 1), offered little separation, while a value at or above the threshold identified a subset with a markedly higher observed arrhythmia burden. This negative predictive value is, however, partly a function of the relatively low outcome prevalence in the cohort (15.2%) and should not be read as an intrinsic property of the marker independent of the population in which it is applied. Whether this distinction can be used prospectively, for example to guide the intensity of follow-up or to prioritize patients for closer surveillance, cannot be determined from a cross-sectional sample and would require confirmation in a longitudinal cohort. If such confirmation is obtained, MHR could be integrated into routine device clinic workflow at essentially no additional cost, because the index is generated automatically from the complete blood count and lipid panel already obtained at follow-up visits. A value at or above a validated threshold could then serve as a simple flag for intensified surveillance—for example, shorter intervals between device interrogations, prioritization for remote-monitoring enrolment, re-evaluation of the adequacy of guideline-directed heart failure therapy, and attention to modifiable inflammatory and metabolic risk factors—whereas a value below the threshold, in view of the high negative predictive value observed here, might support standard follow-up intervals. Any such threshold-guided strategy would, however, need to be tested prospectively before clinical adoption. What can be said now is that, because MHR is generated as a by-product of tests already ordered in essentially every ICD candidate, any prospective role it may ultimately have would impose no incremental cost or patient burden, an advantage over imaging-based or specialized biomarker strategies [7,16].

Whether modifying the balance between monocyte-driven inflammation and HDL’s anti-inflammatory and antioxidant reserve, through lipid-directed, anti-inflammatory, or lifestyle interventions, alters arrhythmic risk cannot be inferred from an observational association and remains a question for future interventional study.

Several features strengthen these findings. Outcome adjudication was blinded to laboratory values, the sample and event count were adequate for the models fitted, and the analysis went beyond conventional reporting to include internal validation (Section 3.6), which indicated that the model was unlikely to be overfitted and that its predicted probabilities were reliable, an aspect of biomarker performance that is not always assessed. The association also persisted after adjustment for statin therapy and amiodarone use (Section 3.4), and MHR itself was uncorrelated with statin use, arguing against confounding by lipid-lowering therapy as an explanation for the findings.

This study also has limitations. Its cross-sectional design relates MHR to arrhythmia already documented at the same assessment and therefore establishes association rather than temporal prediction; whether MHR forecasts future arrhythmic events can only be determined prospectively. We wish to emphasize this point: adequately powered, prospective, ideally multicentre studies with predefined follow-up and prespecified arrhythmic endpoints are needed to establish the predictive—rather than merely associative—value of MHR, including its incremental value over established risk markers, before any clinical application can be recommended. The study was conducted at a single center without external validation, and the derived threshold, although internally stable, requires confirmation in independent cohorts before clinical adoption. The cohort was predominantly male (78%), reflecting the typical demographic of ICD populations, and whether the association holds specifically in women warrants separate confirmation. The outcome combined non-sustained and sustained ventricular arrhythmia; NSVT and device-treated sustained VT/VF differ in clinical significance, and this composite should therefore be interpreted as capturing a broad arrhythmogenic phenotype rather than a single clinical entity of uniform severity. We did not analyze ICD shock burden or arrhythmia frequency as graded outcomes because these data were not available in the present dataset, which is itself an important direction for future work. Detailed ICD programming characteristics, including detection-zone cut-offs, were not systematically recorded and could not be analyzed; heterogeneity in device programming across patients could in principle have influenced which episodes were captured as events. MHR and the comparator markers were measured at a single time point, so we could not examine whether temporal changes carry additional information. Finally, as an observational study it cannot establish causality, and the association between MHR and the arrhythmic substrate should be regarded as hypothesis-generating; direct confirmation would require correlation with tissue-level or imaging measures of fibrosis, such as late gadolinium enhancement or T1 mapping on cardiac magnetic resonance imaging, which were beyond the scope of this analysis and are a natural focus for future studies combining MHR with cardiac imaging biomarkers.

## 5. Conclusions

MHR is independently associated with ventricular arrhythmia in ICD recipients and identifies a threshold that meaningfully separates patients with a lower versus higher observed arrhythmia burden, regardless of cardiomyopathy etiology. Because it is calculated from tests performed routinely in every ICD candidate, MHR is an attractive low-cost candidate for further evaluation in risk stratification. Prospective, multicentre studies with external validation, longitudinal follow-up, and graded arrhythmic outcomes are warranted to determine whether MHR predicts future events and to define how it might be integrated into clinical decision-making.

## Figures and Tables

**Table 1:** Baseline characteristics of the study cohort stratified by the ventricular arrhythmia outcome.

Variable	No Arrhythmia (*n* = 324)	Arrhythmia (*n* = 58)	*p*
Age, years	66 (60–72)	70 (64–73)	0.028
Male sex, *n* (%)	253 (78.1)	44 (75.9)	0.839
LVEF, %	32.5 (30–38)	30 (25–35)	0.172
Ischaemic etiology, *n* (%)	212 (65.4)	43 (74.1)	0.252
CRT-D (vs. conventional ICD), *n* (%)	74 (22.8)	4 (6.9)	0.004 *
Secondary prevention indication, *n* (%)	116 (35.8)	30 (51.7)	0.031
Hypertension, *n* (%)	201 (62.0)	53 (91.4)	<0.001
Diabetes mellitus, *n* (%)	104 (32.1)	36 (62.1)	<0.001
Atrial fibrillation history, *n* (%)	67 (20.7)	16 (27.6)	0.316
Beta-blocker, *n* (%)	296 (91.4)	57 (98.3)	0.118
ACEI/ARB/ARNI, *n* (%)	276 (85.2)	58 (100.0)	0.004
MRA, *n* (%)	161 (49.7)	43 (74.1)	0.001
Amiodarone, *n* (%)	60 (18.5)	7 (12.1)	0.316
White-cell count, ×10^3^/µL	6.9 (5.5–8.0)	8.4 (6.7–10.2)	<0.001
Neutrophils, ×10^3^/µL	4.0 (3.2–4.8)	5.2 (4.4–6.4)	<0.001
Lymphocytes, ×10^3^/µL	1.9 (1.6–2.2)	1.8 (1.5–2.4)	0.723
Monocytes, ×10^3^/µL	0.54 (0.46–0.62)	0.76 (0.58–0.85)	<0.001
HDL-cholesterol, mg/dL	44 (39–48)	44 (37–51)	0.993
hs-CRP, mg/L	1.0 (1.0–2.0)	1.0 (1.0–2.8)	0.850
NLR	2.1 (1.6–2.7)	2.8 (2.1–3.9)	<0.001
NT-proBNP, pg/mL	1355 (768–2225)	1507 (547–2891)	0.287
eGFR, mL/min	67 (56–83)	76.5 (59–89)	0.067
MHR	12.3 (10.1–14.6)	16.3 (13.7–21.3)	<0.001

Data are median (interquartile range) or *n* (%). * Fisher’s exact test (applied where an expected cell count was <5); chi-squared test used for all other categorical comparisons. ACEI/ARB/ARNI, angiotensin-converting enzyme inhibitor/angiotensin receptor blocker/angiotensin receptor–neprilysin inhibitor; CRT-D, cardiac resynchronisation therapy defibrillator; HDL, high-density lipoprotein; hs-CRP, high-sensitivity C-reactive protein; LVEF, left ventricular ejection fraction; MHR, monocyte-to-HDL ratio; MRA, mineralocorticoid-receptor antagonist; NLR, neutrophil-to-lymphocyte ratio; NT-proBNP, N-terminal pro-B-type natriuretic peptide; eGFR, estimated glomerular filtration rate.

**Table 2:** Multivariable logistic regression for the ventricular arrhythmia outcome (primary model). Model AUC 0.780 (*n* = 382; 58 events); covariates chosen a priori as described in Section 2.4. CI, confidence interval; eGFR, estimated glomerular filtration rate; LVEF, left ventricular ejection fraction; MHR, monocyte-to-HDL ratio; OR, odds ratio.

Variable	OR	95% CI	*p*
MHR, per unit	1.23	1.16–1.32	<0.001
Age, per year	1.05	1.01–1.09	0.007
LVEF, per %	0.98	0.94–1.03	0.452
Ischaemic etiology	1.66	0.82–3.35	0.158
eGFR, per mL/min	1.02	1.00–1.04	0.024

## Data Availability

The data presented in this study are available on request from the corresponding author. The data are not publicly available owing to privacy and ethical restrictions.

## Abbreviations

The following abbreviations are used in this manuscript:

ACEI	Angiotensin-Converting-Enzyme Inhibitor
ARB	Angiotensin-Receptor Blocker
ARNI	Angiotensin-Receptor–Neprilysin Inhibitor
AUC	Area under the receiver operating characteristic curve
CI	Confidence interval
hs-CRP	High-sensitivity C-reactive protein
HDL	High-density lipoprotein
ICD	Implantable cardioverter-defibrillator
LVEF	Left ventricular ejection fraction
MHR	Monocyte-to-HDL ratio
MRA	Mineralocorticoid-receptor antagonist
NLR	Neutrophil-to-lymphocyte ratio
NPV	Negative predictive value
NT-proBNP	N-terminal pro-B-type natriuretic peptide
OR	Odds ratio
PPV	Positive predictive value
ROC	Receiver operating characteristic
VF	Ventricular fibrillation
VT	Ventricular tachycardia
eGFR	Estimated glomerular filtration rate
